# Neurotoxicity Associated With Cancer Therapy

**DOI:** 10.6004/jadpro.2012.3.1.2

**Published:** 2012-01-01

**Authors:** Eva Lu Lee, Laurel Westcarth

**Affiliations:** From MD Anderson Cancer Center, Houston, Texas

## Abstract

Neurologic complications can result from direct or indirect effects of cancer therapy. Treatment toxicity may affect both the central nervous system and the peripheral nervous system. Early recognition of these toxicities plays an important role in the management of patients with cancer.

Cancer therapy uses a combination of treatment modalities such as surgery, radiation, and chemotherapy that may improve patient prognosis (Van Meir, Bellail, & Phyphanich, 2004; Butowski & Chang, 2005). However, combination therapy and extended survival are often associated with potential acute or delayed toxicities, including neurotoxicities. Patients with tumors of the nervous system are at particular risk for these complications. Traumatic or ischemic injuries are manifestations of central nervous system (CNS) toxicity as a result of brain-directed surgery to treat primary brain tumor or metastatic disease (Chi, Behin, & Delattre, 2008). Neurotoxicity related to chemotherapy is a common complication and often constitutes a dose-limiting toxicity. As newer agents and targeted therapies are developed, the number and range of potential neurotoxicities also increases (Dropcho, 2010a). Exposure of the CNS to therapeutic radiation, whether direct or incidental, is a potential risk for symptomatic neurologic injury (Dropcho, 2010b). Familiarity with the common neurologic toxicities experienced by patients with CNS tumors will assist the advanced practitioner in oncology in timely assessment and effective therapeutic interventions for these patients.

## Central Nervous System

**SURGERY** 

**Figure 1:**
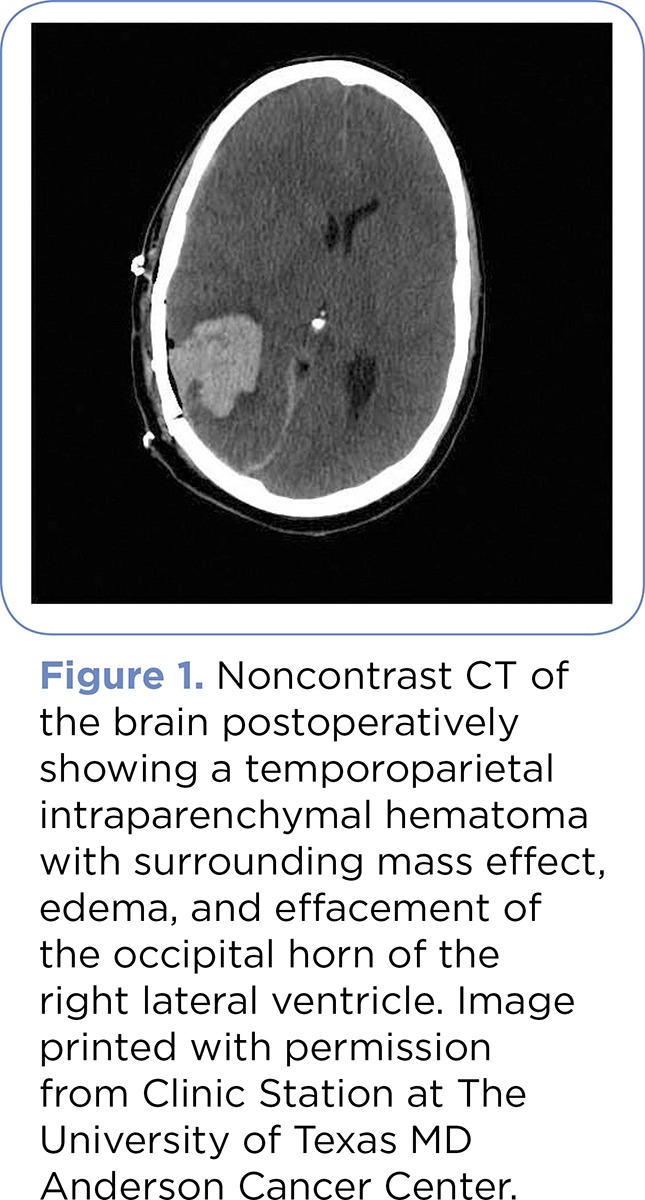
Figure 1. Noncontrast CT of the brain postoperatively showing a temporoparietal intraparenchymal hematoma with surrounding mass effect, edema, and effacement of the occipital horn of the right lateral ventricle. Image printed with permission from Clinic Station at The University of Texas MD Anderson Cancer Center.

Maximum safe resection is the most important goal of surgery for malignant glioma (Chang et al., 2003). This provides histologic diagnosis, reduces neurologic symptoms, and prolongs survival. Complications associated with craniotomy can be classified as neurologic, regional, and systemic (Table 1). Neurologic complications can be a consequence of direct injury to normal brain structures, cerebral edema, vascular injury, or hematoma (Warnick & Petr, 2008; Figure 1). There is also a predisposition to acquire skin organisms and subsequent infection when patients undergo procedures causing barrier breakdown such as brain resection or the placement of shunts, monitoring devices, or ventricular reservoirs (Pruitt, 2008).

The Glioma Outcome Project by Chang et al. studied patients with glioma for presenting symptoms, tumor and patient characteristics, perioperative complications, and neurologic outcomes of first vs. second craniotomies. Headache was more common with first craniotomies, while papilledema and an altered level of consciousness were commonly seen in patients undergoing second surgeries. The risk of perioperative complication was slightly higher for repeated craniotomies than that for the initial surgery (Chang et al., 2003). Regional complications occurred at comparable rates in both groups. Systemic infections occurred more frequently in the repeated craniotomy group.

Sawaya et al. studied neurologic outcomes in patients who underwent craniotomies to remove intra-axial brain neoplasms (gliomas and metastatic tumors), with findings showing that gross total resection can be done in eloquent areas of the brain with an acceptable level of neurologic deficit. Therefore, they concluded that tumors located in eloquent brain regions are not automatically a contraindication for surgical resection (Sawaya et al., 1998; Table 1).

**Table 1:**
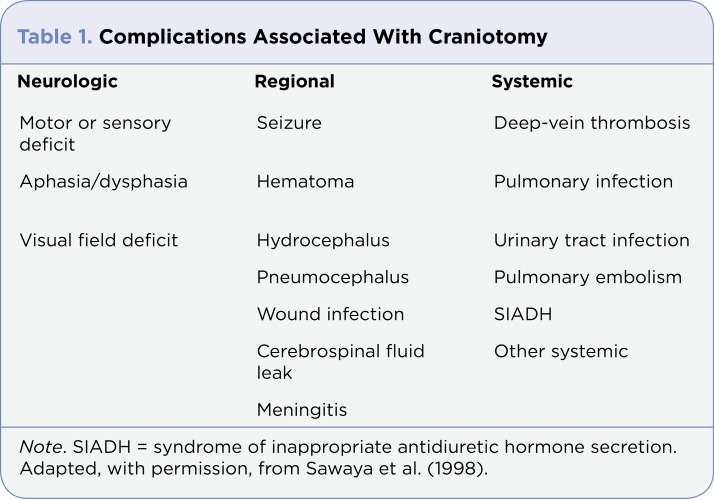
Table 1. Complications Associated With Craniotomy

**Figure 2:**
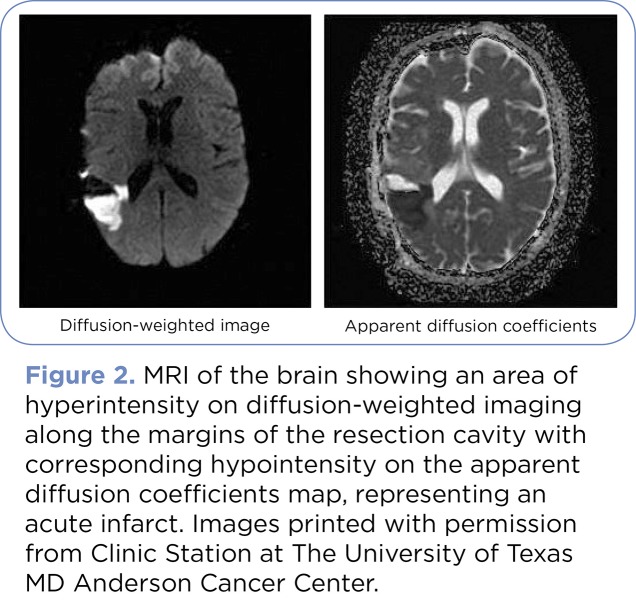
Figure 2. MRI of the brain showing an area of hyperintensity on diffusion-weighted imaging along the margins of the resection cavity with corresponding hypointensity on the apparent diffusion coefficients map, representing an acute infarct. Images printed with permission from Clinic Station at The University of Texas MD Anderson Cancer Center.

New advances in neurosurgical procedure using fusion imaging of three- dimensional magnetic resonance imaging (3D-MRI) and/or three-dimensional computed tomography (3D-CT) with intraoperative MRI during surgery allow the maximal resection possible with preservation of necessary neurologic functions (Wakabayashi et al., 2009). The use of multimodal neuroradiologic imaging in combination with highly technologic analyses is useful for making neurosurgical procedures possible, especially in noncircumscribed brain tumors such as glioma (Wakabayashi et al., 2009). Diffusion-weighted imaging (DWI) is an essential diagnostic tool in the postoperative setting for the early detection of ischemic brain injury that could potentially result in infarction and development of neurologic deficits during surgical resection (Prabhu, Levine, Rao, Shah, & Weinberg, 2009). Several studies have shown that postoperative ischemic brain injury is common in glioma surgical resections and best detected by DWI showing diffusion restriction. Ulmer et al. (2006) studied clinical and radiographic features of peritumoral infarction following surgical resection of glioblastoma and found that focal areas of restricted diffusion adjacent to high-grade glioma surgical cavities were seen in 70% of patients on immediate postoperative MRI studies (Ulmer et al., 2006; Figure 2).

**RADIATION** 

Radiation therapy is widely used and plays a central role in the treatment of primary and metastatic brain tumors (Tofilon & Fike, 2000; Kim, Brown, Jenrow, & Ryu, 2008). It is indicated for curative or palliative intent. Radiation of tumors proximal to normal CNS structures is limited by normal tissue tolerance and the inherent radioresistance of tumor cells; hence, cytotoxic agents and radiosensitizers concurrent with radiation are used to improve tumor control (Belka, Budach, Kortmann, & Bamberg, 2001; Woo & Mahajan, 2008). Radiation toxicity affecting the CNS includes focal cerebral necrosis, neurocognitive deficits, and less commonly, cerebrovascular disease, myelopathy, or the occurrence of a radiation-induced neoplasm (Woo, 2007; Dropcho, 2010b). Cranial nerve involvement is possible in radiation-induced, late-delayed complications if included in the radiation portal. These complications were rare; however, large daily radiation fractions increased this risk (Chi, Behin, & Delattre, 2008). A current study in brain tumor survivors revealed that 17% of patients developed neurosensory impairment and that radiation therapy exposure greater than 50 Gy to the posterior fossa was associated with a greater chance of developing hearing impairment (Chi, Behin, & Delattre, 2008).

A randomized controlled trial by Chang et al. (2003) suggested that patients who underwent stereotactic radiosurgery (SRS) plus whole-brain radiation therapy (WBRT) had more decline in learning and memory function by 4 months than patients that received SRS alone. One possible approach to preserve cognitive function in patients with newly diagnosed brain metastases is an initial therapy with a combination of SRS and close clinical monitoring (Wang et al., 2009). The mechanism of radiation-induced neurotoxicity has been attributed to demyelination or vasculopathy (Farace & Melikyan, 2008). Radiation effects are classified in three phases: acute, early delayed, and late delayed.

*Acute*: The onset of an acute reaction is usually from hours to within 2 weeks after starting cranial radiation therapy, as a result of tumor cell death. Symptoms may include headache, nausea, somnolence, fever, and worsening of preexisting neurologic symptoms (Dropcho, 2010b). Acute toxicity responds well with initiation or increase in steroids, especially in patients with bulky primary or secondary cranial tumors or significant edema at risk for herniation (Asmis, Chung, Teitcher, Kelsen, & Shah, 2008).

*Early Delayed*: Symptoms of early delayed effects occur approximately 4 to 8 weeks after radiation and are attributed partly to temporary disturbance of myelin synthesis due to radiation injury to the oligodendrocytes (Chi, Behin, & Delattre, 2008). Symptoms include somnolence syndrome (drowsiness, lethargy), worsening of preexisting symptoms, or transient cognitive (attention, memory) impairment (Asmis et al., 2008). Steroid therapy is effective and accelerates recovery.

*Late Delayed*: Late delayed effects of radiation appear months to years after radiation is completed. It can manifest as focal brain radionecrosis, which mimics tumor recurrence, cognitive impairment, and leukoencephalopathy, pituitary- hypothalamic dysfunction, or secondary brain tumors (Asmis et al., 2008; Chi, Behin, & Delattre, 2008). These are attributed to vascular endothelial injury and the direct effect of radiation on glial cells (Chi, Behin, & Delattre, 2008).

**TREATMENT RECOMMENDATIONS** 

Agents such as methylphenidate, modafinil, and donepezil have lately been prescribed to improve patients’ cognitive function following radiation treatment for anaplastic glioma or brain metastases (Dropcho, 2010b). Clinical and sometimes radiographic improvement, though temporary, are outcomes of steroid therapy in patients with focal RT necrosis. Anecdotal reports also revealed clinical and radiographic improvement in patients treated with warfarin, hyperbaric oxygen, vitamin E, or pentoxifylline. Improvement has also been reported recently with the use of bevacizumab (Avastin), a monoclonal antibody against vascular endothelial growth factor (VEGF), which acts to decrease vascular permeability and normalize the blood-brain barrier (Dropcho, 2010b).

## Chemotherapy

Chemotherapeutic agents can cause either direct or indirect neurotoxic complications. Encephalopathy, peripheral neuropathy or myopathy, cerebellar dysfunction, and myelopathy which are caused by intrathecal chemotherapy administration are examples of direct manifestation of chemotherapy agents (Chang & Butowski, 2008). The blood-brain barrier, a tight junction of endothelial cells lining blood vessels in the brain, forms a barrier between the circulation and the brain parenchyma. It blocks certain agents from entering the nervous system at a cellular level. The intactness of the blood-brain barrier determines whether or not chemotherapeutic agents will reach the nervous system. In order to bring about a neurotoxic effect, a chemotherapeutic agent must be able to cross the blood-brain barrier. Methotrexate is one of the chemotherapeutic agents that can cross the blood-brain barrier once a certain dose level is reached. Methotrexate at a high dose in the CNS causes encephalopathy or occasionally posterior-reversible encephalopathy syndrome (PRES), which is disscussed below. Symptoms of acute high-dose methotrexate neurotoxicity include somnolence, confusion, and seizures within 24 hours of treatment (Dietrich & Wen, 2008).

**POSTERIOR REVERSIBLE ENCEPHALOPATHY SYNDROME** 

"PRES is an increasingly recognized neurologic disorder with characteristic MRI findings associated with a multitude of diverse clinical entities" (Hodnett, Coyle, O’Regan, Maher, & Fanning, 2009, p. 494). PRES has several etiologies and is also associated with the use of immunosuppressive therapy such as tacrolimus and cyclosporine as well as other agents such as cisplatin, rituximab (Rituxan), erythropoietin, and bevacizumab. Common presenting clinical findings in PRES include headache, mental status changes, focal neurologic deficits, and visual changes. The main symptoms observed were encephalopathy (92%), seizure (87%), headache (53%), and visual symptoms (39%; Hodnett et al., 2009). PRES is a condition with a high morbidity and mortality rate because it is not quickly recognized.

**Figure 3:**
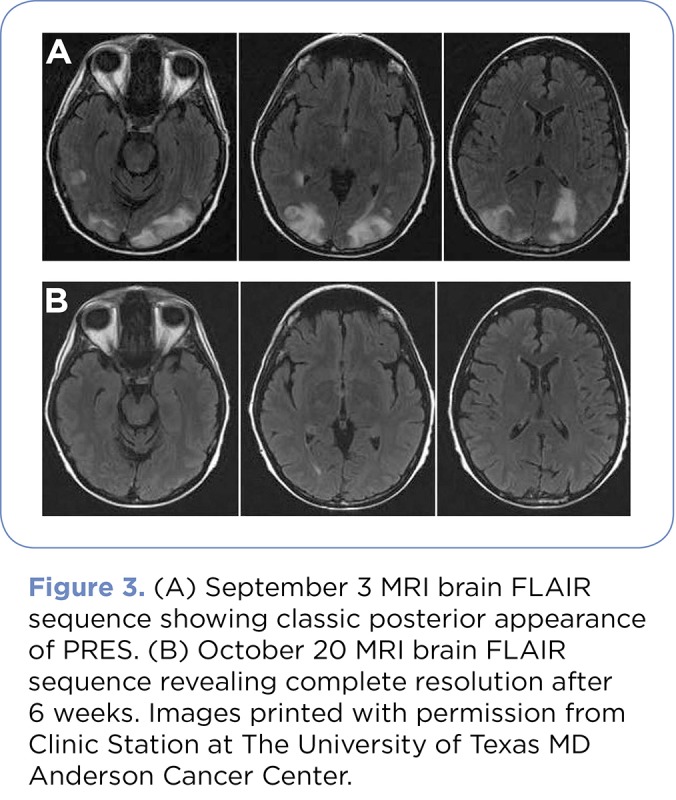
Figure 3. (A) September 3 MRI brain FLAIR sequence showing classic posterior appearance of PRES. (B) October 20 MRI brain FLAIR sequence revealing complete resolution after 6 weeks. Images printed with permission from Clinic Station at The University of Texas MD Anderson Cancer Center.

In addition to clinical and hematologic findings, imaging is warranted in order to confirm a PRES diagnosis. Although CT of the brain is utilized as diagnostic imaging, MRI of the brain is the most sensitive imaging for the diagnosis of PRES. "PRES appears on MRI as symmetrical, subcortical white matter and gray matter lesions on FLAIR and T2-weighted sequences predominantly located posteriorly" (Hodnett et al., p. 494). Changes in the subcortical region are said to be due to reversible vasogenic edema (Figure 3).

**TREATMENT RECOMMENDATIONS** 

Early recognition of clinical and neuroradiologic manifestation is essential in the management of PRES. PRES is a reversible condition that warrants either a dose reduction or withholding of the causative agent, which will result in a complete recovery for most patients (Aranas, Prabhakaran, & Lee, 2009; Hodnett et al., 2009). If blood pressure is elevated in a patient with PRES, it should be lowered gradually; a rapid blood pressure reduction can increase the size of the involved ischemic area (de Oliveira et al., 2008). Seizures deserve particular attention and must be promptly treated with antiepileptic drugs (Striano et al., 2005). Intravenous anticonvulsant agents are used to control seizures and are preferred because they can be rapidly loaded. The goal is to prevent further neurotoxic damage in this reversible condition. Follow-up MRI should be performed.

**INTRACRANIAL/INTRACEREBRAL HEMORRHAGE** 

Intracranial hemorrhage (ICH), an indirect vascular neurotoxic complication of cancer (Chang & Butowski, 2008), is the result of ruptured cerebral vessels leading to the development of a hematoma in the brain parenchyma (Xue, Hollenberg, & Yong, 2006). Intracranial hemorrhage into the brain occurs more often in patients with metastatic tumors than in those with primary brain tumors (Rogers, Leary, & Saver, 2008). Melanoma and lung cancer with brain metastasis are two cancers that are commonly associated with ICH, as well as metastatic renal, thyroid, and germ cell tumors. Glial tumors are the most common CNS neoplasms associated with ICH. Patients commonly present with headache, nausea, vomiting, obtundation, and seizures. "Histologic factors associated with intratumoral hemorrhage include rapid tumor growth, tumor necrosis, vessel thrombosis, the presence of multiple thin- walled vessels and tumor invasion of adjacent cerebral vessels" (Rogers, Leary, & Saver, p. 216).

Thrombocytopenia due to chemotherapy can lead to intracranial hemorrhage. Non–small cell lung cancer (NSCLC) and breast cancer patients with brain metastasis have a high predisposition for spontaneous cerebral hemorrhage. Patients with CNS metastases due to certain malignancies such as hepatocellular carcinoma and renal cell cancer have a higher propensity for ICH (Khasraw, Holodny, Goldlust, & Deangelis, 2011).

Intracranial hemorrhage occurs in PRES; however, studies that focus on this subgroup of the population that is affected are limited (Hefzy, Bartynski, Boardman, & Lacomis, 2009). PRES-related cerebral hemorrhage is caused by thrombocytopenia and other abnormal coagulation disorders. PRES-related hemorrhage is also common in patients who are post bone marrow transplant on cyclosporine and tacrolimus for the prevention of graft-vs.-host disease.

According to the side-effect profile, bleeding in the CNS may occur in patients on bevacizumab, a systemic cancer agent approved for recurrent glioblastoma, metastatic colorectal, and non–small cell lung cancers. In spite of the fear of ICH with the use of bevacizumab, retrospective analysis has revealed a low rate of spontaneous ICH even in the presence of CNS metastasis (Khasraw et al., 2011).

MRI and CT are both used to evaluate ICH. "CT findings suggestive of tumoral hemorrhage include early edema and an indentation appearing on the hematoma surface on noncontrast studies, which demonstrates after contrast injection enhancement, while ICH on MRI is that of signal heterogeneity, evidence of nonhemorrhagic tumor mass, delayed hemorrhage evolution, hemosiderin deposition, and early edema" (Rogers, Leary, & Saver, p. 217).

**TREATMENT RECOMMENDATIONS** 

Treatment of ICH is directed toward the underlying cause of the hemorrhage, e.g., correction of an underlying coagulopathy, whole-brain radiation, or systemic chemotherapy for hyperleukocytosis and intracranial leukemic filtration (Chamberlain, 2010). Patients with leukemia and encephalopathy warrant evaluation for disseminated intravascular coagulation and should receive a coagulopathy screening (Chamberlain, 2010; Khasraw et al., 2011). Concurrent coagulopathy may increase the risk of bevacizumab-related ICH.

**SEIZURES** 

Seizures are common neurologic complications in cancer patients. They can occur as a result of structural abnormalities of the brain (brain metastasis), cerebrovascular disease, reversible posterior leukoencephalopathy syndrome, and radiation toxicity (Pulzova, Bhide, & Andrej, 2009). Seizures can also occur as a result of metabolic impact of the cancer or cancer treatment (Ashkenazi et al., 2010). The following chemotherapeutic agents are known to cause seizures: amifostine, asparaginase, BCNU, busulfan, chlorambucil, cisplatin, cyclophosphamide, cytosine arabinoside, dacarbazine, docetaxel, etanercept (Enbrel), etoposide, 5-FU, gemcitabine, hexamethylamine, hydroxyurea, ifosfamide, interferon, Il-2, letrozole, leuprolide, levamisole, mechlorethamine, methotrexate, octreotide, paclitaxel, pentostatin, suramin, temozolomide, teniposide (Vumon), thalidomide (Thalomid), and vinca alkaloids (Glantz & Batten, 2008).

Seizures are much more common with tumors located in the supratentorial regions than in the infratentorial regions of the brain; among the supratentorial tumors, seizures are much more frequent with superficial and cortical lesions (63% of cases) than with tumors situated within the basal ganglia or entirely in the white matter (29% of cases; Bonoiu et al., 2009). More than 50% of glioma patients will experience seizure recurrence during the course of their illness, while 11% with brain metastases and 19% with neoplastic meningitis will experience recurrent seizures during their considerably shorter life-spans (Glantz & Batten, 2008).

**TREATMENT RECOMMENDATIONS** 

A diagnostic evaluation is required to determine the cause of seizures. Epilepsy treatment can be divided into several categories: the use of antiepileptic drugs, surgical resection of the epileptic foci and other surgical measures, the removal of factors causing and precipitating the event, and the regulation of bodily and mental activity. However, the use of antiepileptic drugs is the most key aspect of treatment. In just about 70% of all patients with epilepsy, seizures are controlled completely or nearly completely with antiepileptic drugs (Ropper et al., 2005); see Table 2. Careful consideration should be made as to the selection of a particular antiepileptic drug (AED) because many of the widely used AEDs induce the cytochrome P450 (CYP450) enzymes CYP3A4, CYP2C8, and CYP2C9, causing significant drug interactions in patients receiving chemotherapy metabolized by the same enzymes (Batchelor & Byrne, 2008).

**Table 2:**
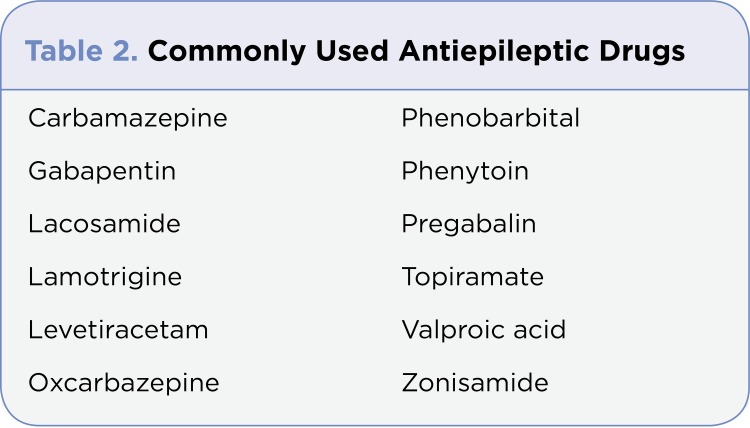
Table 2. Commonly Used Antiepileptic Drugs

**COGNITIVE IMPAIRMENT** 

A decline in neurocognitive and neurobehavioral functioning during the acute phase of treatment has been linked with adjuvant chemotherapy; however, it is not known whether or not, or to what level, these symptoms continue. Potential neurologic complications of chemotherapy include acute encephalopathy (confusional state, insomnia, agitation), chronic encephalopathy (cognitive dysfunction consistent with subcortical dementia, incontinence, gait disturbance), stroke-like episodes, and cerebellar symptoms (Farace & Melikyan, 2008). The development of neurocognitive and neurobehavioral alteration produced by radiation follows an expected pattern, and the time and path of their development are associated with treatment parameters, adjuvant therapy, and patient characteristic. The incidence of radiation-induced neurocognitive changes is best studied in whole-brain radiotherapy, whereas the effects of stereotactic radiosurgery and intensity-modulated radiotherapy have not yet been comprehensively studied (Farace & Melikyan, 2008).

**TREATMENT RECOMMENDATIONS** 

At the present time, there are no proven treatments for cognitive impairment following brain cancer and subsequent cranial irradiation, nor are there any known effective preventive strategies. Methylphenidate has been used to treat many symptoms associated with advanced cancer, including cancer-related fatigue, opioid-induced sedation, depression, and cognitive dysfunction associated with malignancies. Modafinil, another psychostimulant, after its approval for use in attention deficit disorder in 1998, is now increasingly used for cancer-related symptoms (Farace & Melikyan, 2008). Evaluating patients to determine preexisting cognitive deficits is vital to evaluate the results of treatment and provide a baseline for assessment of the true effect of therapy in assessing cognitive decline (Iuvone et al., 2011).

## Peripheral Nervous System

**PERIPHERAL NEUROPATHY** 

"Peripheral neuropathy is defined as any injury, inflammation, or degeneration of the peripheral nerve fibers" (Armstrong, Almadrones, & Gilbert, 2005). Peripheral neuropathy is estimated to occur in 10% to 20% of patients with cancer. It is usually associated with the use of platinum drugs, taxanes, epothilones, vinca alkaloids, and newer agents such as bortezomib (Velcade) and lenalidomide (Revlimid; Fernandez- de-las-Penas et al., 2010). Chemotherapy-induced peripheral neurotoxicity (CIPN) is a common and dose-limiting side effect of cancer therapy. Symptoms of CIPN can be severe and incapacitating; as they progress, they can hinder the patient from receiving optimal therapy (Pachman, Barton, Watson, & Loprinzi, 2011). In patients with cancer, peripheral neuropathy symptoms range from mild paresthesias to severe motor neuropathy, which can cause immobility. Paclitaxel, cisplatin, vincristine, oxaliplatin, and bortezomib are chemotherapy agents that cause dose- limiting peripheral neuropathy. Of all the chemotherapy agents, vincristine is the major culprit of early peripheral neuropathy, while cisplatin is known to cause delayed symptoms that are evident several months after the discontinuation of the drug. Vincristine and paclitaxel affect both small and large nerve fibers while cisplatin affects predominantly large fibers (Armstrong, Almadrones, & Gilbert, 2005).

Loss of deep tendon reflexes and paresthesia are the two most common and earliest symptoms of peripheral neuropathy caused by vincristine. The incidence of areflexia due to vincristine is 57% while that of digital paresthesia is about 23% to 36%. However, many of the neuropathies caused by vincristine are slowly reversible, with some symptoms resolving quickly while others are delayed. Paresthesia is the most quickly reversible symptom, with either a dose reduction or cessation of the causative agent. On the other hand, loss of deep tendon reflexes is considered one of the slower symptoms to resolve.

There are some elements of CIPN that remain unsolved despite its clinical relevance in that it minimizes the potential use of antineoplastic agents and newer cancer therapies. One aspect is how to assess the occurrence and severity of CIPN in the most dependable and effective way (Cavaletti et al., 2010). We have limited knowledge of the underlying mechanisms of CIPN; however, structural properties of the different compounds that are neurotoxic might contribute to the difference in pathogenic mechanisms of injury, in addition to the form of neurotoxicity, severity of the clinical condition, and incidence of CIPN.

In summary, symptoms associated with peripheral neuropathy include sensory loss in stocking-glove distribution, loss of position sense, and vibration (large fiber); stocking-glove distribution and loss of pain and temperature sensation (small fiber); loss of deep tendon reflexes and foot drop (motor); and constipation, obstipation, ileus, and fluctuations in blood pressure (autonomic).

**TREATMENT RECOMMENDATIONS** 

In the presence of CIPN, dose reduction or discontinuation of the causative agent is usually required. There are no effective methods or drugs available that can prevent or ameliorate the long-term side effects of CIPN. Another goal of treatment is to relieve pain; some of the common medications used are anticonvulsants such as gabapentin, topiramate, carbamazepine, phenytoin, and pregabalin; and tricyclic antidepressant medications such as amitriptyline and nortriptyline, and the serotonin and norepinephrine reuptake inhibitor duloxetine. The topical analgesics amitriptyline, ketamine, and/or baclofen have been studied for the treatment of neuropathic pain. No significant systemic absorption or toxicity was associated with topical application; this potentially offers an advantage over oral preparations, particularly in a patient population prone to polypharmacy and systemic toxicity (Dropcho, 2010a).

Calcium and magnesium (Ca/Mg) infusions have been well-studied treatments for the prevention of oxaliplatin-associated CIPN (Pachman et al., 2011). Several data suggest that vitamin E may also diminish the development of CIPN, but more data regarding its effectiveness and safety should be obtained before recommending general use in patients. Other agents that are being investigated and look promising in preliminary studies, but need substantiation, include glutathione, N- acetylcysteine, oxcarbazepine, and xaliproden. "Effective treatment of established CIPN, however, has yet to be found" (Fernandez-de-las-Penas et al., 2010). Other agents used are glutamine, an amino acid with a neuroprotective effect, and alpha- lipoic acid (thioctic acid), a potent lipophilic antioxidant effective in the treatment of diabetic neuropathy.

**MYOPATHY** 

Steroid therapy is the mainstay of treatment for cerebral edema, but it is associated with a myriad of adverse effects. One of the most common neurotoxicities attributed to this medication is proximal myopathy. Fluorinated glucocorticoids such as dexamethasone are more likely to cause myopathy than nonfluorinated glucocorticoids like prednisone or hydrocortisone (Kesari, Paleologos, & Vick, 2008). Although corticosteroids have varied benefits in both liquid and solid tumors, they can lead to a variety of systemic and neurologic complications. Myopathy, which manifests as skeletal muscle weakness and tenderness, is a common side effect and toxicity associated with steroid therapy (Owczarek, Jasi?ska, & Orszulak-Micalak, 2005). Myopathy may be so severe that it can impair mobility by preventing ambulation and causing an inability to lift the arms or legs. Ten to twenty percent of patients will develop steroid myopathy within the first 2 weeks of steroid therapy while 65% will develop this condition within 12 weeks of treatment (Chamberlain, 2010). Subsequently, patients should be maintained on the lowest possible dose that controls neurologic complications.

**TREATMENT RECOMMENDATIONS** 

Recovery from myopathy due to steroids may take several months; therefore, medication reduction and cessation should be initiated immediately, along with exercise or physical therapy (Kesari, Paleologos, & Vick, 2008; Chamberlain, 2010).

**MYELOPATHY** 

Toxicity of cancer therapy can cause reversible and irreversible damage to the CNS (Schlegel, 2011). "Toxicity to the spinal cord is rare but severe and most frequently it is the result of intrathecal drug administration" (Schlegel, 2011, p. 25). Methotrexate and liposomal cytarabine are two agents that are known to cause irritation of the spinal cord and subsequent spinal cord dysfunction. Due to direct irritation to the spinal cord, patients commonly experience transient pain that may ultimately progress to spinal cord dysfunction. Acute myelopathy with paresis, although rare, is a devastating complication of intrathecal chemotherapy caused by these agents (Schlegel, 2011).

Myelopathy not only occurs as a consequence of intrathecal therapy, but also as a complication of radiation therapy to the spinal cord. Epidural spinal cord metastasis is a common complication that occurs in 5% to 8% of all patients with cancer (Schlegel, 2011). It begins as a pain syndrome that progresses to myelopathy. Radiation myelopathy may present as early as 4 to 6 months or as late as 1 to 2 years after radiation treatment (Dropcho, 2010b). Early myelopathy, the most common form of radiation myelopathy, appears as a transient paresthesia with sensory level that gradually resolves over several months. Delayed radiation myelopathy symptoms may wax and wane from several months to as long as 10 years. Unlike the symptoms of early radiation myelopathy, delayed myelopathy patients report less pain and lower extremity dysesthesias, weakness, and sphincter dysfunction. In 50% of patients these neurologic symptoms gradually progress to paraplegia or quadriplegia. Once ambulation is lost, it is rare for it to return.

Evidence of spinal cord abnormality is visible on images of patients with delayed radiation myelopathy. "MRI imaging may reveal widening of the affected cord and abnormal signal intensity on T2-weighted images with abnormal intramedullary contrast enhancement, either in a streaky or less commonly a ring-enhancing pattern" (Dropcho, 2010b, p. 225). Persistent spinal cord atrophy will be visible on images of the long-term survivors.

Radiation myelopathy may occur even if the dose of radiation is considered safe or standard. However, the risk of delayed radiation myelopathy increases with higher total RT (radiation therapy) dose or a larger daily fraction. Incidentally, only 0.5% of the patients who receive the standard radiation dose (4,500 to 5,000 cGy) and 5% of the patients who receive a total dose of 5,700 to 6,100 cGy experience delayed radiation myelopathy. Delayed radiation myelopathy may start immediately or, more frequently, in a progressive manner; patients present with sensory and/or motor deficits leading to para- or tetraparesis. A characteristic initial clinical presentation is a Brown-Sequard syndrome, consisting of a motor deficit which is an ipsilateral hemiplegia with contralateral pain and temperature sensation deficits. Some patients develop a transverse myelopathy with bilateral leg weakness and sensory loss up to the irradiated region. Other patients experience pain, bladder, and bowel sphincter as well as diaphragmatic dysfunction in upper cervical spinal cord lesions (Chi, Behin, & Delattre, 2008).

**TREATMENT RECOMMENDATIONS** 

Early diagnosis is imperative in both late and delayed radiation myelopathy since treatment is limited and this condition can lead to paralysis (Dropcho, 2010b). Treatment with corticosteroids may provide stabilization or partial improvement; however, patients often become steroid-dependent. Other regimens that may be of some benefit include warfarin or hyperbaric oxygen. There is no existing proven long-term management for delayed radiation myelopathy (Chi, Behin, & Delattre, 2008).

**PLEXOPATHY** 

Radiation neurotoxicity can affect any part of the neuroaxis, including the brachial and lumbar area (Quant & Wen, 2010b). Most importantly, radiation neurologic complications should be clearly differentiated from other pathologies such as compression due to tumor infiltration. Whether the plexopathies are caused by direct tumor infiltration or as a late effect of radiation therapy, early recognition and evaluation is warranted (Chamberlain, 2010). Dysesthesias and lymphedema are associated with radiation plexopathy, whereas pain, lower plexus involvement, and Horner’s syndrome are characteristic of infiltrative cancer (Chamberlain, 2010).

Radiation brachial plexopathy is most common in breast cancer, followed by lung cancer and lymphoma (Dropcho, 2010b). The etiology is postulated to be due to extensive fibrosis within and surrounding nerve trunks of the plexus, and demyelination and loss of axon were evidenced at surgery and autopsy. However, the exact cause of radiation-induced brachial plexopathy is unclear. "RT injury to the lumbosacral plexus or cauda equina most commonly occurs after treatment of pelvic tumors, testicular tumors or tumors involving para-aortic lymph nodes" (Dropcho, 2010b, p. 227). RT lumbosacral plexopathy may occur as early as a few months to up to 5 years posttherapy. Symptoms are usually consistent with lower extremity weakness, atrophy, fasciculation, areflexia, bowel and bladder symptoms, and pain. Diagnostic workup for plexopathies should include needle electromyography, PET, CT, and MRI (Dropcho, 2010b).

**TREATMENT RECOMMENDATIONS** 

Physical therapy may be beneficial to maintain muscle strength. Warfarin was reported to be of benefit to some patients with both lumbar and brachial plexopathies (Dropcho, 2010b). It is important to recognize this condition early in order to target therapy and interventions.

## ACKNOWLEDGMENT

The authors thank Dr. Marta Penas-Prado, assistant professor of Neuro-Oncology at The University of Texas MD Anderson Cancer Center, for her invaluable assistance and fruitful discussion.
